# Assessment of the impact of acute inflammation on the anti-cancer responses in a mouse model

**DOI:** 10.1186/2051-1426-1-S1-P267

**Published:** 2013-11-07

**Authors:** Sohaila M Galal, Mohamed L Salem, Zeinab A Ibrahim

**Affiliations:** 1 Zoology, Faculty of Science, Tanta, Egypt

## Background

Inflammation is a vital process induced by microbial infection or tissue injury. The main function of inflammation is to resolve the infection or repair the damage and return the body to a state of homeostasis. Acute inflammation can induce signals that result in two main functions: 1) initiation of an inflammatory cascade that helps limits the infection and 2) activation of the immune response. In this study we determined the impact of the nature and magnitude of acute inflammation on the host anti-tumor activity against Ehrlich Ascites cancer cell (EAC) line as a model system which forms either peritoneal ascites upon intraperitoneal injection.

## Design

Different inflammatory signals, including agonists for toll-like receptor (TLR) 2 (zymosan) and TLR3 agonist (poly (I:C) or its clinical grade Hiltonol®), complete Freund's adjuvant (CFA), and incomplete Freunds Adjuvant (IFA), and BCG from Bacillus calmatte (TLR9 agonist) were used to induce acute inflammation 1 day after i.p. challenge with EAC.

## Results

We found that Hiltonol®, poly (I:C), IFA, CFA, and BCG induced inflammatory cells associated with anti-tumor activity that resulted in significant decrease in the tumor growth measured by the total number of tumor cells after 7 days of tumor challenge. Of interest, among the test TLR agonists both poly(I:C) and Hiltonol® (TLR3 agonist) showed the highest anti-tumor effects.

## Conclusions

Provision of the proper inflammatory signal with optimally defined magnitude and duration during cancer growth will induce inflammatory cells with potent anti-tumor responses leading to significant decreases in tumor growth.

